# Children

**Published:** 1891-03

**Authors:** 


### CHILDREN.

If you would see a woman or a child graceful, beautiful, and charming, you must find one that is loved. The child that dreads to be corrected or criticised for every word or movement never has a manner of elegance or an expression of charm. Fill your child's soul with an ideal of good manners, of benevolence and beauty; teach it to dislike vulgarity, selfishness, rudeness, and to feel that you love and adore it, and expect of it charming manners, and the work is accomplished. It is impossible for a slave to have any style. If you would have your child dignified, you must treat it with dignity. It is wrong to correct a child in public. Any proud child feels degraded by it. It should be a case of dire necessity when you find fault with a child before strangers; to destroy a child's pride is to do him an irreparable injury. Take advantage of some intimate hour when parent and child are alone together, and then let the parent tenderly explain how the child has behaved ill the day before or that morning, and why its conduct was wrong, and how it should have behaved; it shows that the parent respects it and loves it, and believes in its capacity to do all good things. This will have ten times the effect of punishment, when the child is in a state of excitement and the parent usually angry. Get in the habit of explaining the reason of things to your child. Let there be as little confusion in its mind as possible. Above all, keep the fact of your love uppermost in the child's mind, and let it understand that you have no wish to domineer over it, only that, being older and wiser, and loving the child so much, you would save it from its inexperience, that this is your duty, that you are teaching it to be its own master. If your child is cross, do not punish him, but distract his mind from the subject that annoys him. If he continues to be cross, suspect his stomach, and assure yourself that this is in perfect order; a troubled digestion is the root of bad temper.

